# Mistaken Diabetic Ulcers: A Case of Bilateral Foot Verrucous Carcinoma

**DOI:** 10.1155/2018/4192657

**Published:** 2018-01-23

**Authors:** Vanessa Di Palma, Jill P. Stone, Andrew Schell, Jeffrey C. Dawes

**Affiliations:** ^1^Department of Obstetrics and Gynecology, University of Calgary, Calgary, AB, Canada; ^2^Division of Plastic Surgery, Department of Surgery, University of Calgary, Calgary, AB, Canada; ^3^Department of Pathology & Laboratory Medicine, University of Calgary and Calgary Laboratory Services, Calgary, AB, Canada

## Abstract

Verrucous carcinoma (VC) is a rare, low-grade, and well-differentiated variant of squamous cell carcinoma. These tumors are slow-growing and exophytic and have a negligible incidence of metastasis. Treatment is complete surgical resection, ideally by Mohs micrographic surgery, to ensure adequate clear margins. Cutaneous VC predominantly occurs on the plantar surface of the foot and rarely occurs in multiple sites. This case study describes the fourth reported occurrence of bilateral VC of the feet in a woman with chronic diabetic foot ulcers. The case provides further support for persistent wounds contributing to the development of this lesion and describes their role in the characteristic delay in diagnosis of VC.

## 1. Introduction

Verrucous carcinoma (VC) is a rare, low-grade, and well-differentiated variant of squamous cell carcinoma (SCC) first described by Ackerman in 1948 [1]. The tumors are slow-growing and exophytic, eventually developing a bulky polypoid, cauliflower-like appearance [2]. These broad based tumors can ulcerate and may have sinus tracts that drain foul-smelling keratin or purulent material [2]. Although locally deeply invasive, compressing but not destroying underlying soft tissue, VC tumors show minimal dysplasia and have a negligible incidence of metastasis [2, 3]. Diagnosis requires deep biopsy, and treatment is complete surgical resection, ideally by Mohs micrographic surgery, to ensure adequate clear margins.

VC usually occurs in the oropharynx, genitalia, and feet [4]. Typically presenting in the 5th to 6th decade, VC is five times more likely to occur in men than women [3, 5]. Cutaneous VC is referred to as epithelium cuniculatum and predominantly occurs on the plantar surface of the foot [2]. Epithelium cuniculatum rarely occurs in multiple sites and is almost always unilateral [6]. This case study describes the fourth reported occurrence of bilateral epithelium cuniculatum of the feet and illustrates several challenges typical of diagnosis and treatment of VC.

## 2. Case Report

**Figure 1:**
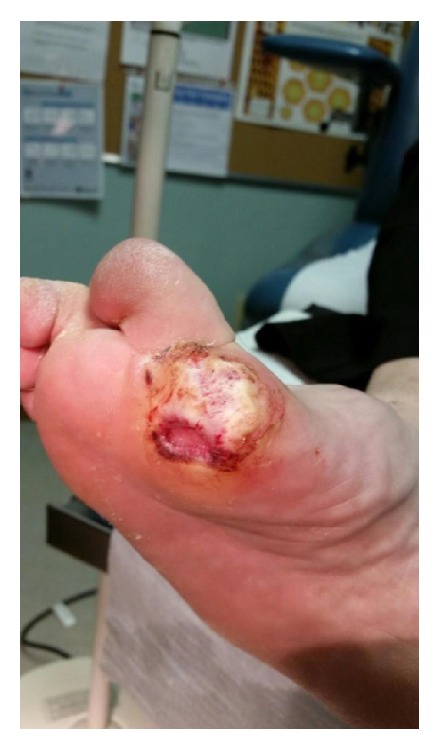
Exophytic lesion at the base of the first MTP joint of the right foot within a chronic diabetic ulcer. Pathology confirmed this lesion to be verrucous carcinoma.

This case involves a 44-year-old woman with uncontrolled type 2 diabetes and severe peripheral neuropathy in both feet. In late 2012, during routine follow-up for her right foot chronic ulcers, a growth was found underlying the plantar aspect of her right first metatarsal phalangeal (MTP) joint. She described this lesion as “sharply painful”; her other ulcers caused her a “more dull pain.” Eventually, the growth became cauliflower-like and drained a pungent, toothpaste-like substance.  Figure 1 shows this exophytic lesion within a diabetic ulcer prior to excision. A wide excision of this lesion was performed by a dermatologist in October 2013. Pathology confirmed a diagnosis of VC. The patient was followed up closely by dermatology and podiatry. In December 2014, a biopsy was performed in the area of her previous VC showing recurrence of the disease. She was referred to another dermatologist for confirmation of this diagnosis versus one of Charcot arthropathy. Mohs resection of the lesion was performed in March 2015, with pathology confirming VC. Podiatry concluded that difficulties with wound healing in the area of the first MTP were likely due to VC recurrence rather than Charcot arthropathy.

**Figure 2:**
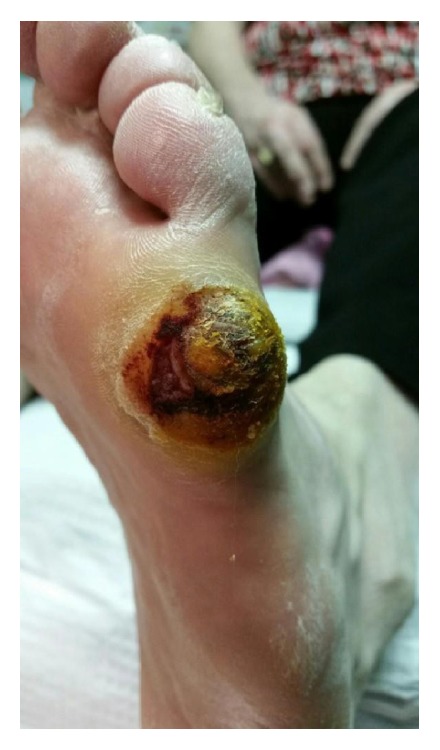
Painful exophytic lesion on the plantar surface of the left 5th MTP joint, which, after multiple biopsies, was confirmed by pathology to be verrucous carcinoma.

**Figure 3:**
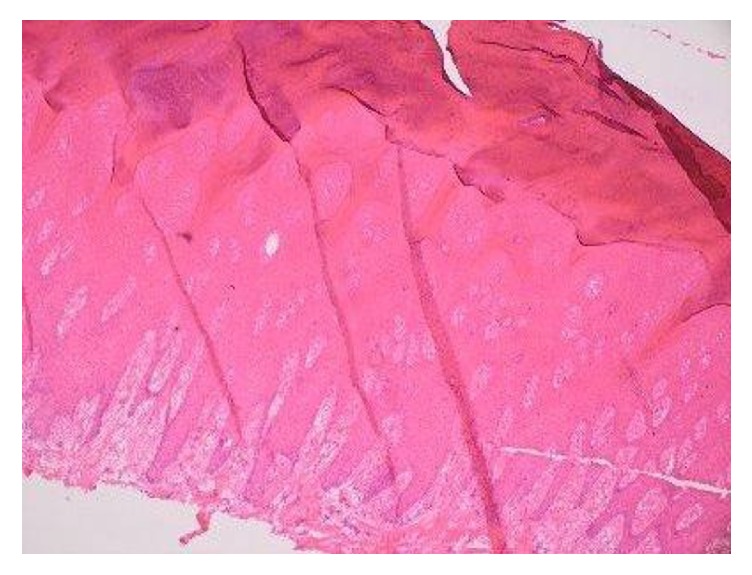
Representative medium power image of the verrucous carcinoma demonstrates prominent surface hyperkeratosis and marked epidermal acanthosis, with a combined exophytic and endophytic growth pattern. Cytologic atypia is only mild to moderate. Rather than the infiltrative pattern typically seen at the deep edge of conventional invasive squamous cell carcinoma, verrucous carcinoma demonstrates insidious pushing-type invasion by expansile and irregular rete pegs (4x, H&E stain).

Throughout early 2015, the patient and her home care team noted an acutely painful lesion on her left foot on the plantar surface of the 5th MTP joint (Figure 2). The patient suspected VC; however, due to the rare nature of VC tumor, clinical suspicion was low for bilateral disease. Unfortunately, the patient was not seen for this lesion again until May 2015, when an incisional biopsy was taken of this growth. Pathology confirmed VC; however, as the biopsy was small and the base of the lesion was not visualized, two more biopsies of the lesion were collected between May and July of 2015, all showing VC. Mohs resection of the tumor was performed, showing complete resection of the lesion.  Figure 3 shows the left foot after Mohs resection of this lesion. In November 2015, the patient's right second toe was amputated due to complications from her ulcers. Pathology described a prominent verrucous epithelial hyperplasia, with focal atypia that approaches but is not diagnostic of VC.

## 3. Discussion

Verrucous carcinoma rarely involves multiple sites [7]. This is the fourth described case of bilateral disease and describes disease at multiple sites on the same foot. This case also details the characteristic delay in diagnosis of VC and the requirement for aggressive surgical excision to ensure complete resection.

The presence of concomitant diabetic ulcers likely contributed to both the bilateral nature and delayed diagnosis of the tumor. Long-term irritation and trauma have both been implicated in increasing likelihood of tumor occurrence [2, 8]. In several cases of VC, the tumor was reported to arise from preexisting lesions with chronic inflammation, such as chronic ulcers. Chronic wounds are associated with weight-bearing areas, which are also are more frequently affected by VC than non-weight-bearing areas [3, 9]. The patient reported that although the initial presentation of the VC was visually very similar to a diabetic ulcer, the pain she experienced was quite different. She stated that as months went on, the lesion differentiated itself by its whitish “cauliflower-like” appearance and foul-smelling exudate. This distinction is important, as the patient also had infected ulcers and was familiar with draining wounds.

Human papillomavirus (HPV) types 6, 11, 16, and 18 have been found to colocalize with various types of VC3, with HPV 6 and 11 being most commonly associated with those occurring in the anogenital region [2]. Despite some specimens displaying oncogene expression or altered p53 activity, many authors discovered no evidence of HPV DNA in VC specimens. Interestingly, a 2012 case study of bilateral VC of the feet did report expression of HPV 16 in the resected specimen [7]. It could be informative to analyze the current samples for tumor suppressor mutations, such as activating mutations in p53, which may suggest the involvement of an oncogenic virus in carcinogenesis [4, 5]. Further, future analysis of normal and VC tissue may display the expression of specific genetic markers that put patients at higher risk of this disease.

The patient endured many biopsies and incomplete resections prior to her Mohs surgeries, which were successful in obtaining clear margins. Mohs technique has been reported to effectively allow total tumor removal while preserving the maximum normal tissue; however, amputation is sometimes necessary when the tumor is too extensive, as in this case [9, 10]. This case also demonstrates the need for aggressive resection rather than small local excisions for both diagnosis and cure. Due to the insidious presentation of this tumor, time to diagnosis is often delayed, during which the patient suffers pain and disability. Knowledge of the obscurities and pathogenesis of VC can help lead the clinician to accurate diagnosis and treatment for the patient.
