# Corrigendum to “Mistaken Diabetic Ulcers: A Case of Bilateral Foot Verrucous Carcinoma”

**DOI:** 10.1155/2018/6405129

**Published:** 2018-04-15

**Authors:** Vanessa Di Palma, Jill P. Stone, Andrew Schell, Jeffrey C. Dawes

**Affiliations:** ^1^Department of Obstetrics and Gynecology, University of Calgary, Calgary, AB, Canada; ^2^Division of Plastic Surgery, Department of Surgery, University of Calgary, Calgary, AB, Canada; ^3^Department of Pathology & Laboratory Medicine, University of Calgary and Calgary Laboratory Services, Calgary, AB, Canada

In the article titled “Mistaken Diabetic Ulcers: A Case of Bilateral Foot Verrucous Carcinoma” [1], the first affiliation is incorrectly linked to the fourth author. The corrected affiliation is shown above.
